# Tadalafil Ameliorates Chronic Ischemia-Associated Bladder Overactivity in Fructose-Fed Rats by Exerting Pelvic Angiogenesis and Enhancing p-eNOS Expression

**DOI:** 10.3390/ijms26031363

**Published:** 2025-02-06

**Authors:** Wei-Chia Lee, Steve Lu, Chia-Hao Su, You-Lin Tain, Kay L. H. Wu, Chien-Ning Hsu, Hong-Tai Tzeng

**Affiliations:** 1 Division of Urology, Kaohsiung Chang Gung Memorial Hospital, Chang Gung University College of Medicine, Kaohsiung 833, Taiwan; dinor666@ms32.hinet.net; 2 Institute for Translational Research in Biomedicine, Kaohsiung Chang Gung Memorial Hospital, Kaohsiung 833, Taiwan; leu@cgmh.org.tw (S.L.); wlh0701@yahoo.com.tw (K.L.H.W.); 3 Department of Biotechnology, College of Life Science, Kaohsiung Medical University, Kaohsiung 807, Taiwan; 4 Center for General Education, Chang Gung University, Taoyuan 333, Taiwan; chiralsu@gmail.com; 5 Department of Radiation Oncology, Kaohsiung Chang Gung Memorial Hospital, Kaohsiung 833, Taiwan; 6 Department of Biomedical Imaging and Radiological Sciences, National Yang Ming Chiao Tung University, Taipei 112, Taiwan; 7 Department of Pediatrics, Kaohsiung Chang Gung Memorial Hospital, Chang Gung University College of Medicine, Kaohsiung 833, Taiwan; tainyl@hotmail.com; 8 Department of Pharmacy, Kaohsiung Chang Gung Memorial Hospital, Kaohsiung 833, Taiwan; cnhsu@cgmh.org.tw; 9 School of Pharmacy, Kaohsiung Medical University, Kaohsiung 807, Taiwan

**Keywords:** angiogenesis, bladder, fructose, ischemia, tadalafil

## Abstract

Metabolic syndrome (MetS) can contribute to a chronic ischemia-relative overactive bladder (OAB). Using fructose-fed rats (FFRs), a rat model of MetS, we investigated the effects of tadalafil (a phosphodiesterase-5 inhibitor) on MetS-associated chronic bladder ischemia and bladder overactivity. Phenotypes of the OAB, including increased micturition frequency and a shortened intercontractile interval in cystometry, were observed in FFRs, together with reduced bladder blood perfusion (in empty bladders) via laser color Doppler imaging and elevated serum nitrite levels, suggesting chronic ischemia-related bladder dysfunction. Treatment with tadalafil (2 mg/kg) promoted pelvic angiogenesis, as shown by magnetic resonance imaging, and increased VEGF and p-eNOS overexpression in the bladder. This treatment restored bladder perfusion and alleviated bladder overactivity without significantly altering most MetS parameters. At the molecular level, FFRs exhibited increased ischemia markers (NGF, HIF-2α, and AMPK-α2) and decreased p-AMPK-α2, along with elevated proinflammatory mediators (ICAM-1, nuclear NF-κB, COX-2, IL-1β, IL-6, and TNF-α), enhanced mitochondria biogenesis (PGC-1α, TFAM, and mitochondria DNA copy number), oxidative stress (decreased nuclear NRF2, increase MnSOD and 8-OHdG staining), and tissue fibrosis (increased TGF-β1, collagen I, and fibronectin). Tadalafil treatment improved these effects. Together, these findings suggest that tadalafil may promote VEGF-associated angiogenesis, enhance p-eNOS staining in the bladder vasculature, normalize bladder perfusion in microcirculation, and reduce serum nitrite levels. Consequently, tadalafil mitigates the adverse effects of chronic ischemia/hypoxia, improving bladder overactivity. We elucidated the mechanisms underlying the tadalafil-mediated amelioration of MetS-associated OAB symptoms.

## 1. Introduction

An overactive bladder (OAB) is characterized by urinary urgency as its core feature, often accompanied by frequency, nocturia, and urgent incontinence, with a prevalence of 19-31% in adults [1,2]. OAB significantly affects quality of life, impairs social interactions, disrupts sexual activity, and may lead to depression [3,4]. Metabolic syndrome (MetS), a prediabetic condition, comprises cardiovascular risk factors such as insulin resistance, central obesity, hypertension, hypertriglyceridemia, and hypercholesterolemia. Its prevalence reaches 41% in developed countries like the United States [5] and can exceed 30% in urban populations of emerging Asian economies [6], presenting a growing public health concern. Emerging evidence suggests that MetS can be associated with OAB by sharing common pathophysiologies [7,8]. Furthermore, MetS-associated OAB is a notable subtype that often does not respond well to standard antimuscarinic therapies [9].

Preclinical and clinical studies suggest that MetS may lead to OAB through chronic pelvic ischemia, given its cardiovascular risk factors [10,11,12,13,14,15]. Azadzoi et al. simulated chronic bladder ischemia in a rabbit model by inducing endothelial injury with an arterial balloon and a 0.5% cholesterol diet, observing resulting bladder overactivity, fibrosis, oxidative stress, and neurodegeneration [12,13]. A meta-analysis by Gacci et al. found an association between the severity of lower urinary tract symptoms (LUTSs) and major adverse cardiac events [14]. The arterial supply to the urinary bladder originates from internal iliac arteries, with these bifurcations often being key sites for atherosclerosis [10]. Papaefstathiou et al. used endovascular revascularization to recanalize the common iliac artery in patients with steno-occlusive disease, alleviating LUTS severity and improving bladder and erectile function [15].

Tadalafil, a selective phosphodiesterase type 5 (PDE5) inhibitor, increases NO/cyclic guanosine monophosphate signaling, leading to smooth muscle relaxation [16]. Initially used to treat erectile dysfunction, 5 mg daily of tadalafil is now a first-line treatment for LUTS secondary to benign prostatic hyperplasia (European Urological Association guidelines) [17]. Tadalafil also plays a role in managing OAB [9,16]. A randomized clinical trial by Chen et al. demonstrated its effectiveness in reducing OAB symptom scores in women [18]. The proposed mechanisms of action include detrusor muscle relaxation, improved bladder oxygenation, vasodilation, and reduced oxidative stress [16,19]. However, the precise mechanisms underlying tadalafil’s benefits in bladder ischemia-associated OAB require further investigation.

Epidemiological studies show a correlation between rising global fructose consumption and MetS prevalence [20]. The fructose-fed rat (FFR) model, therefore, serves as an appropriate animal model for MetS-associated bladder dysfunction [21]. High fructose intake induces metabolic disturbances, including insulin resistance, hypertension, dyslipidemia, and bladder overactivity, mimicking human MetS [22,23]. This study investigated the hypothesis that tadalafil improves MetS-associated bladder overactivity in FFRs by targeting chronic bladder ischemia, thereby affecting vasculature, bladder perfusion, inflammation, mitochondrial biogenesis, oxidative stress, and bladder remodeling.

## 2. Results

### 2.1. General Characteristics, Micturition Behavior, Bladder Perfusion, and Pelvis Vasculature Among Groups

**Figure 1:**
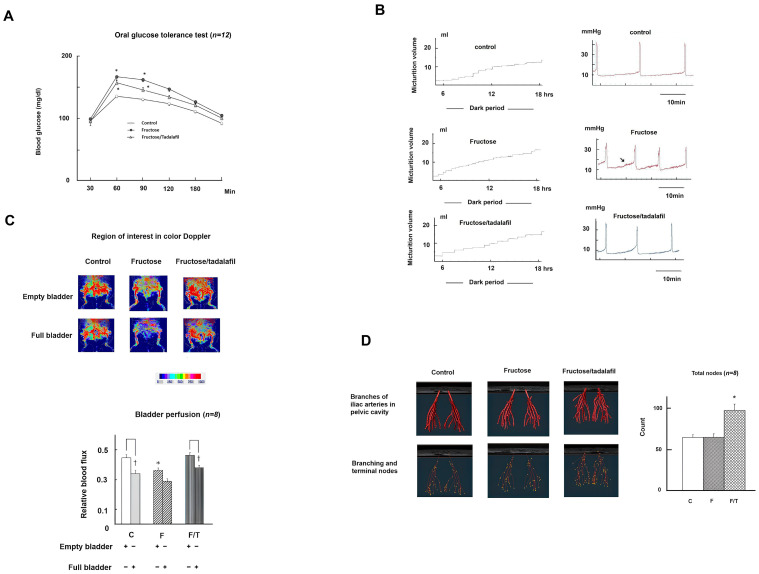
The assessment of glucose tolerance, micturition behavior, bladder blood perfusion, and pelvic vasculature of 3D magnetic resonance imaging in experimental animals. (**A**) Plasma glucose response curves from the oral glucose tolerance test. (**B**) Results from the metabolic cage study (left) and filling cystometry (right) and (**C**) regions of interest are shown in laser color Doppler imaging (upper half) and relative blood flux in both empty and full bladders (bottom half) across groups. (**D**) The reconstruction of pelvic vessels using 3D magnetic resonance imaging (left) and angiogenesis analysis indicating total nodes (right). Data are expressed as mean ± SEM, with *n* = 12 or 8 animals per group. * *p* < 0.05 in comparison with the control group by one-way ANOVA with Dunnett’s test, and ^†^ *p* < 0.05 in paired *t*-test. The arrow indicates the increased basal tone in the panel (**B**).

Table 1 and Figure 1A,B present the general characteristics, micturition behavior, and biochemical results of all experimental groups (*n* = 12). No adverse events were observed. Compared to controls, the fructose-fed group exhibited significantly higher mean values for MetS traits, including the oral glucose tolerance test (OGTT) glucose levels (Figure 1A), mean arterial pressure, serum triglycerides, cholesterol, uric acid, and insulin, as well as an increased homeostasis model assessment of insulin resistance. FFRs also displayed the phenotypes of bladder overactivity, characterized by increased micturition frequency in metabolic cage studies, shortened intercontractile intervals, non-voided contractions, and an increased basal detrusor tone in cystometry (Figure 1B). Tadalafil treatment significantly alleviated bladder overactivity in FFRs and restored serum nitrite levels without affecting MetS parameters. Laser Doppler imaging (Figure 1C) revealed significantly reduced bladder blood flow influx in FFRs (empty bladder), which tadalafil restored. MRI analysis (Figure 1D) revealed a significant increase in angiogenesis, quantified as the sum of branching and terminal nodes (vascular density), in tadalafil-treated FFRs.

### 2.2. The Expressions of Phosphorylated-Endothelial Nitric Oxide Synthase (p-eNOS), Ischemia Markers, and Proinflammatory Molecules of the Bladder

**Figure 2:**
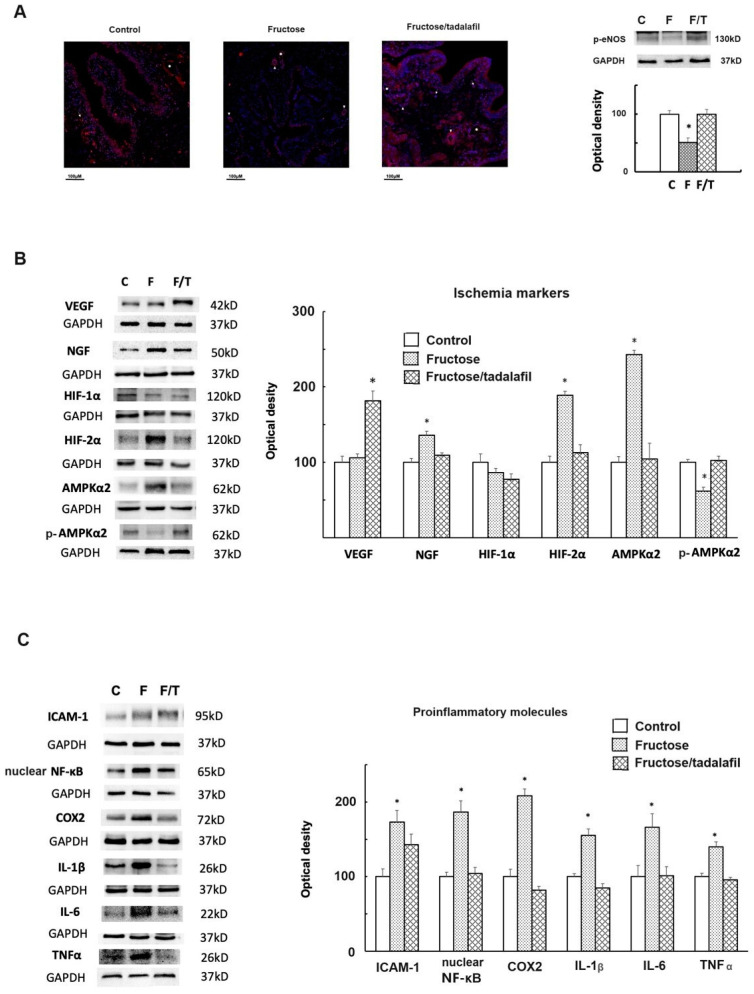
p-eNOS staining and Western blot analysis of ischemia markers and proinflammatory molecules across groups. (**A**) Immunofluorescence staining of p-eNOS in bladder tissues (reduced from ×400) (left). Arrows indicate capillaries, arrowheads indicate arterioles, and asterisks indicate venules. An abundance of vessels was observed in the submucosal area of tadalafil-treated bladders. Western blot analysis (right) demonstrates a significant decrease in p-eNOS expression in the fructose group. (**B**) Western blot analysis of VEGF, NGF, HIF-1α, HIF-2α, AMPK-α2, and p-AMPK-α2. (**C**) Western blot analysis of ICAM-1, nuclear NF-κB, COX-2, IL-1β, IL-6, and TNF-α. * *p* < 0.05 in comparison with the control group by one-way ANOVA with Dunnett’s test in Western blot analysis.

Figure 2A shows significantly reduced p-eNOS expression in the bladders of FFRs, as demonstrated by both immunofluorescence and Western blotting. Immunofluorescence staining also revealed abundant vessels (arterioles, venules, and capillaries) in the submucosa of tadalafil-treated FFR bladders, suggesting angiogenesis. Figure 2B shows an increased expression of nerve growth factor (NGF), hypoxia-inducible factor (HIF)-2α, and AMPKα2, along with decreased p-AMPKα2 in FFR bladders; tadalafil treatment normalized these ischemia markers and increased vascular endothelial growth factor (VEGF) expression. Figure 2C demonstrates an increased expression of proinflammatory molecules (i.e., (ICAM)-1, nuclear factor (NF)-κB, cyclooxygenase (COX)-2, interleukin (IL)-1β, IL-6, and tumor necrosis factor (TNF)-α) in the FFR group, which was absent in the tadalafil-treated group.

### 2.3. Alterations in Mitochondrial Biogenesis, Oxidative Stress, and Bladder Fibrosis

**Figure 3:**
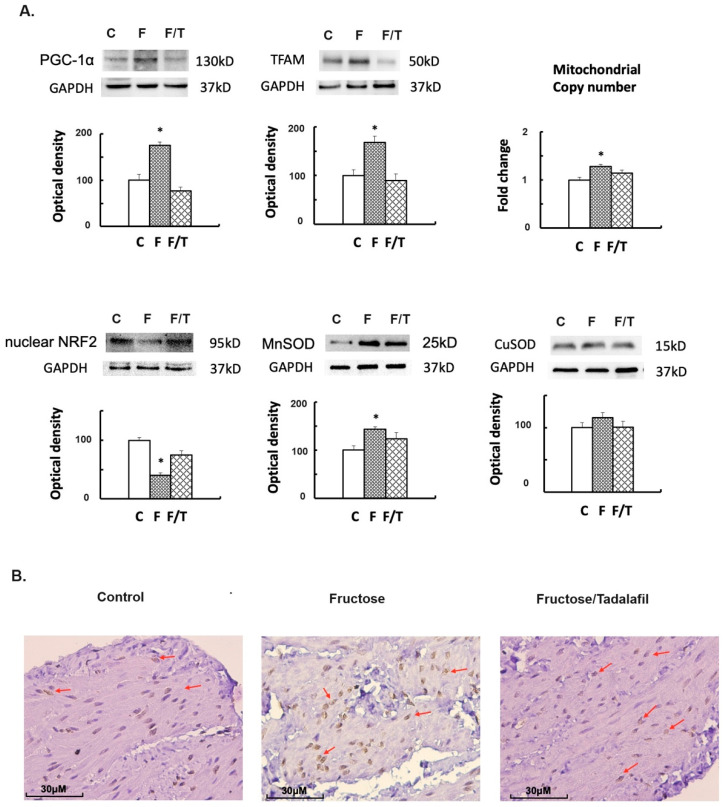
Mitochondrial DNA copy number, Western blot analysis of mitochondrial biogenesis and oxidative stress, and 8-OHdG immunochemistry staining in bladder tissues. (**A**) Western blot analysis of PGC-1α, TFAM, nuclear NRF2, MnSOD, and CuSOD, along with the polymerase chain reaction assessment of the mitochondrial DNA copy number. (**B**) Immunochemistry staining for 8-OHdG (Reduced from ×400). Arrows indicate positive nuclear staining. The fructose group displayed a cluster of positive nuclear staining in the detrusor muscle. * *p* < 0.05 when compared to the control group, as determined by one-way ANOVA with Dunnett’s test in Western blot analysis.

**Figure 4:**
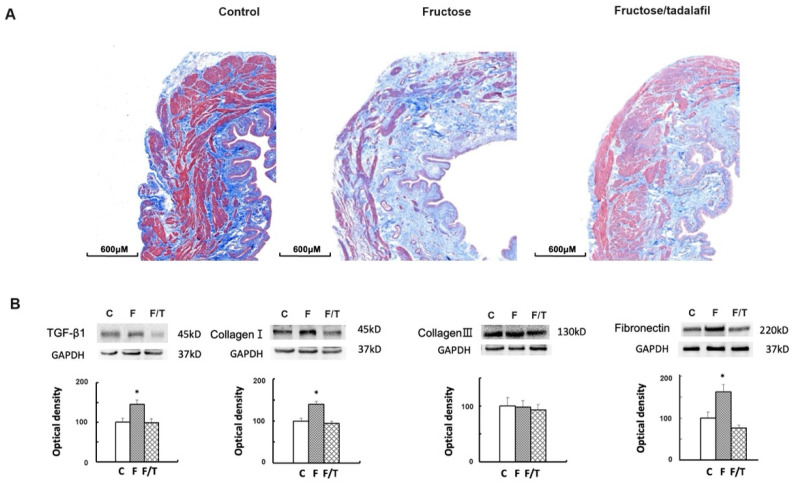
Masson trichrome staining and fibrosis marker analysis in bladder samples. (**A**) Representative image of Masson trichrome staining (Reduced from ×40). (**B**) Western blot analysis assessing the expression of TGF-β1, collagen I, collagen III, and fibronectin. * *p* < 0.05 compared to the control group, determined by one-way ANOVA with Dunnett’s test.

FFRs exhibited an increased expression of peroxisome proliferator-activated receptor gamma coactivator (PGC)-1α and mitochondrial (mt) transcription factor A (TFAM) expressions, as well as the mtDNA copy number (Figure 3A). Simultaneously, decreased nuclear factor erythroid 2-related factor 2 (NRF2) and increased Mn superoxide dismutase (SOD) were observed in FFR bladders; tadalafil treatment reversed these alterations. Immunohistochemistry (IHC) staining for 8-hydroxy-2′-deoxyguanosine (8-OHdG) revealed nuclear 8-OHdG staining clustered in the detrusor muscle of FFRs, which was attenuated by tadalafil treatment (Figure 3B). Furthermore, in the Masson trichrome stain, the FFR’s bladder illustrates submucosal bladder fibrosis (Figure 4A). For fibrogenesis markers, the fructose group showed a significant increase in transforming growth factor (TGF)-β1, collagen I, and fibronectin (Figure 4B). However, no significant change in fibrogenesis markers was observed in the tadalafil-treated group.

## 3. Discussion

**Figure 5:**
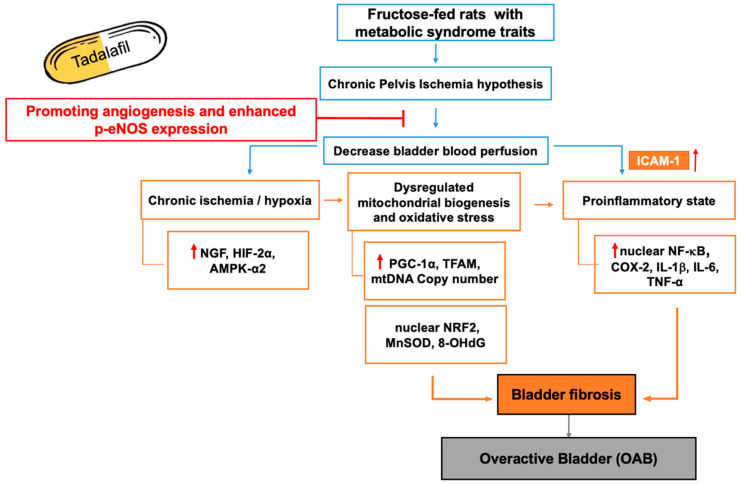
Schematic diagram illustrating the study hypothesis and effects of tadalafil for treating bladder ischemia-related bladder dysfunction.

This study demonstrates that a high-fructose diet induces chronic bladder ischemia in rats, leading to bladder overactivity. Tadalafil treatment reversed these effects (Figure 5). Elevated serum nitrite, reduced bladder perfusion (empty bladder), and decreased p-eNOS expression in the FFR bladder suggest that chronic bladder ischemia and subsequent hypoxia contribute to bladder dysfunction. Molecularly, FFR bladders showed increased NGF, HIF-2α, and AMPK-α2 expression, along with decreased p-AMPKα2, indicating chronic ischemia. A proinflammatory state was evidenced by increased ICAM-1, NF-κB, COX-2, IL-1β, IL-6, and TNF-α expression. Furthermore, chronic bladder ischemia resulted in dysregulated mitochondrial biogenesis (increased PGC-1α, TFAM, and mtDNA copy numbers) and oxidative stress (decreased nuclear NRF2, increased MnSOD, and increased 8-OHdG staining). Finally, bladder fibrosis, indicated by Masson’s trichrome staining and increased TGF-β, collagen I, and fibronectin, was observed. Tadalafil treatment stimulated angiogenesis (increased MRI vascular density) along with increased VEGF and p-eNOS expression, which restored bladder perfusion in microcirculation and normalized the molecular alterations associated with chronic bladder ischemia.

Our study presents a novel rat model of chronic bladder ischemia induced by a high-fructose diet, eliminating the need for artificial arterial injury. Normal bladder function involves the storage phase (99.7% of the day) and the voiding phase [24]. A full-bladder states, or straining to void, can reduce bladder wall oxygen tension, potentially causing ischemia followed by reperfusion upon voiding—a cyclic ischemia–reperfusion pattern observed in patients with bladder outlet obstruction [10,16]. Clinically, chronic bladder ischemia can arise from vasculopathy-related pelvic ischemia or cyclic ischemia–reperfusion induced by benign prostatic hyperplasia [10]. This study revealed consistently low bladder perfusion and elevated serum nitrite (a marker of hypoxia) in FFRs, suggesting that bladder ischemia/hypoxia is analogous to vasculopathy-related pelvic ischemia, lending support to insufficient p-eNOS activity to convert excess nitrite to NO [22,25,26]. However, tadalafil treatment appeared to mitigate this condition [27,28].

Hypoxia triggers increased oxygen demand and HIF-mediated responses to restore oxygen homeostasis; HIF-1α is associated with acute hypoxia, while HIF-2α is associated with chronic hypoxia [29]. In our FFR model of bladder ischemia, the upregulation of HIF-2α and NF-κB, but not HIF-1α, was observed alongside VEGF expression, suggesting a predominant role for the chronic hypoxia pathway [29]. Christiaansen et al. reported higher HIF-2α expression in primary cultured bladder urothelial cells from OAB patients compared to controls [30]. This may involve HIF-2α-inducible gene suppression (i.e., decreased nuclear NRF2) and NF-κB-mediated proinflammation [29]. Increased responsive proinflammatory mediators (NGF, ICAM-1, COX-2, IL-1β, IL-6, TNF-α) likely contribute to bladder overactivity [31,32]. During ischemia, stress response molecules are activated to mitigate energy deprivation. AMPKα2, abundantly expressed in the bladder and responsive to ischemia [31,33], was overexpressed while its phosphorylated form (p-AMPKα2) was reduced in FFRs. As reported by Yang et al., this dysregulation of AMPKα2 post-translational modification contributes to increased detrusor contractility and nerve fiber loss [33]. AMPKα2 impairment also might integrate stress response signaling in the mitochondria [31,34].

As Yang et al. reported, chronic ischemia/hypoxia can impair bladder mitochondrial function in rats [35]. Hypoxia increases oxidative stress, reduces energy production, and alters mitochondrial morphology [34]. HIF-2α positively correlates with PGC-1α expression in most tissues [36]; PGC-1α, highly expressed in energy-demanding tissues, regulates oxidative metabolism and mitochondrial biogenesis [34,36]. Increased PGC-1α and TFAM expression, along with evidence of increased mitochondrial biogenesis in this study, suggests metabolic stress and redox sensing in ischemic bladder tissue [34,37]. Free radical accumulation and mitochondrial dysfunction have been reported in FFR bladders [38]. Decreased NRF2 and increased MnSOD expressions indicate an attempt to counteract oxidative stress [31,35]. Increased 8-OHdG staining confirms oxidative damage to nuclear and mitochondrial DNA [19,39]. Consequently, bladder ischemia increased TGF-β1, collagen I, and fibronectin expression, resulting in bladder fibrosis in FFR bladders [10]. Our findings corroborate with Matsuo et al.’s report that tadalafil improves OAB symptoms and urodynamic parameters by reducing oxidative stress [19].

As our results show, tadalafil treatment did not significantly alter most MetS parameters in FFRs. However, it stimulated pelvic angiogenesis in the MRI image, as evidenced by increased VEGF, enhanced p-eNOS staining dense vasculature in the bladder, and decreased serum nitrite levels. This resulted in improved bladder perfusion in microcirculation, reduced inflammation, restored mitochondrial biogenesis, mitigated oxidative stress, and lessened bladder fibrosis resulting from chronic ischemia. While PDE5 inhibitors primarily relax smooth muscles via increased cGMP, VEGF-mediated angiogenesis (e.g., sildenafil) and enhanced NO production (via p-eNOS) are also anticipated mechanisms for improved organ perfusion [16,27,28,40]. While eNOS-derived NO is a vasodilator [26], it also provides vasoprotection; Glushakova et al. demonstrated that, in another FFR model, fructose intake suppressed p-eNOS and NO production, and NO donor supplementation reduced surface adhesion molecule (e.g., ICAM-1) expression, preventing leukocyte adhesion to the vasculature [41].

### 3.1. Limitations and Future Prospects

This study has limitations. First, in the treatment of MetS-associated bladder overactivity, mirabegron, a β3-adrenergic agonist, may enhance cAMP levels in the bladder and improve metabolic outcomes in animal models [42]. Comparing the therapeutic potential and off-target effects of mirabegron and tadalafil in the FFR model would be of interest. However, in adherence to the 3R principle for the ethical use of laboratory animals, mirabegron treatment was not included in this study. Second, although tadalafil has been widely used for various pathological conditions, it is not currently recommended as a first-line treatment for OAB in clinical guidelines. The underlying pathology of OAB is highly heterogeneous, with contributing factors such as bladder ischemia, MetS, aging, psychological stress, and alterations in the urinary microbiome [43]. In the era of precision medicine, future research may refine the target population for tadalafil treatment in OAB by considering factors such as age, body mass index, and biomarkers (e.g., oxidative stress and inflammatory cytokines) to optimize therapeutic outcomes [19,44,45]. Furthermore, while antimuscarinic agents remain a common treatment for OAB, they are often associated with adverse effects such as dry mouth and constipation [46]. A study by Yamanishi et al. demonstrated that combining tadalafil with mirabegron resulted in improved symptom control in men with persistent OAB, with minimal adverse effects [47]. This finding suggests that combination therapy may offer a promising alternative approach for managing OAB.

### 3.2. Conclusions

In this study, we demonstrated increased VEGF-associated angiogenesis via MRI and enhanced p-eNOS staining in bladder vasculature, normalized bladder perfusion in microcirculation, and reduced serum nitrite levels, suggesting that tadalafil reversed the chronic bladder ischemia in FFRs. Our findings also elucidate the pathogenesis of the MetS-associated bladder overactivity induced by fructose, revealing the underlying roles of chronic ischemia and subsequential inflammation, mitochondrial biogenesis dysregulation, oxidative stress, and bladder fibrosis. Tadalafil, without significantly altering most MetS parameters, directly restored bladder perfusion, thereby mitigating the negative effects of chronic ischemia/hypoxia and improving bladder overactivity in FFRs. These results support further investigation into the therapeutic potential of PDE5 inhibitors for MetS-associated bladder dysfunction.

## 4. Materials and Methods

### 4.1. Animals

This study was conducted in accordance with the guidelines of the National Research Council, USA. The experimental protocol was approved by the Institutional Animal Ethics Committee (Permit Number: 2020061803). Every effort was made to minimize the suffering of the animals and the number of animals used in our experiments. Sixty female Wistar rats (BioLASCO Taiwan Co., Ltd., Taipei, Taiwan; weight: 200–240 g) were randomly allocated to 3 groups (*n* = 20) and subjected to an experimental course of 12 weeks. They were maintained in a facility accredited by the Association for Assessment and Accreditation of Laboratory Animal Care International under a controlled temperature (24 °C ± 0.5 °C) and a light–dark cycle of 12 h each. For the experiments, rats were divided into 3 groups, namely the control group (regular chow), the fructose group (fructose group; fructose-rich diet; 60% fructose diet, Harlan Teklad, Madison, WI, USA), and the fructose plus tadalafil group (tadalafil group; tadalafil 2 mg/kg, fed by gavage for four weeks beginning at the start of the 9th week) [23].

### 4.2. Metabolic Cage Study and OGTT

At the end of week 11, 12 rats from each group were placed in individual metabolic cages (3701M081, Tecniplast, Buguggiate, Italy) equipped with an FT-104 force transducer (iWorx/CB Sciences, Inc., Dover, NH, USA), as previously reported [23,24]. After a 24 h familiarization period, the volume of liquid consumed, micturition frequency, and urine output were measured over three days to determine an average value. Subsequently, an OGTT was performed following an overnight fast.

### 4.3. Measurement of Bladder Perfusion by Using Laser Doppler Blood Flow Imaging

A laser full-field blood flow imager (Moor FLPI, Moor Instruments, Devon, UK) was used to assess bladder perfusion in microcirculation [48], with the right femoral artery flux serving as a contrast. At week 11, eight rats in each group were anesthetized with intramuscular zoletil (50 mg/kg) and were positioned supine with the low abdominal area shaved (region of interest, ROI). The rat’s urethra was cannulated with a Polyethylene-50 tube to either empty or fill the bladder with sterile normal saline to a capacity of 0.8 mL. The laser Doppler source was mounted on a movable rack, precisely 20 cm above the ROI. The laser beam, reflected from moving red blood cells in nutritional capillaries, arterioles, and venules as an estimate of microcirculation, was detected and processed to provide flux units using moorLDI Review V6.2. The flux unit of the main trunk of the right femoral artery was also measured for contrast. The data were collected and presented as a ratio of flux units in the ROI to the femoral artery.

### 4.4. Three-Dimensional Magnetic Resonance Imaging for Pelvis Angiogenesis

The angiogenesis in the pelvic region of rats was evaluated using a 9.4-T horizontal-bore animal MR scanning system (Biospec 94/20, Bruker, Ettingen, Germany). In week 11, eight rats from each group were anesthetized with intramuscular zoletil (50 mg/kg) and had their bladders emptied. Subsequently, the MRI system was utilized for 3D angiographic imaging to obtain baseline images. We employed an optimized imaging sequence to reconstruct 3D images of the pelvic vasculature and processed the 3D MRI data using ParaVision 7 for angiogenesis analysis, quantifying vascular parameters with a diameter of 100 μm to calculate the total number of branching and terminal nodes of the vessels. Vascular density was calculated as the sum of branching and terminal nodes.

### 4.5. Filling Cystometry

Twelve rats in each group were weighed and then anesthetized by the subcutaneous injection of urethane (1.2 g/kg) at the end of the experiment [23,24]. Polyethylene-50 catheters were placed in the left carotid artery to measure arterial pressure using a PowerLab 16S system with a P23 1D transducer (Gould-Statham, Oxnard, CA, USA). Through the urethra, the bladder catheter was connected, using a T-tube, to a pressure transducer and a microinjection pump (CH-4103; Infors, Bottmingen, Switzerland). Room-temperature saline was infused into the bladder at a rate of 0.08 mL/min. Cystometry was recorded using an RS3400 chart recorder (Gould, Cleveland, OH, USA). All the rats were observed for a minimum period of 30 min to ensure that the voiding pattern was stable. Subsequently, reproducible micturition cycles were recorded for 1 h periods and used for evaluation. An overdose of urethane was then injected to sacrifice the rats. Blood samples were collected for biochemical analysis and serum nitrite and nitrate levels using a NOx Colorimetric Assay kit (Cayman, MI, USA).

### 4.6. Urinary Bladder Histological Studies for the Immunofluorescence Staining of p-eNOS, IHC Staining of 8-OHdG, and Masson Trichrome Stain

To characterize the morphological changes in the urinary bladder of rats, the bladder base from each group was fixed in formalin and embedded in paraffin. After obtaining sections of the bladder, the slides were deparaffinized, fixed, and subjected to antigen retrieval. Immunofluorescence staining for p-eNOS was performed by incubating the sections with a primary antibody against p-eNOS (Ser 1175) (1:100 dilution; Affinity) at 25 °C for 1 h, followed by incubation with a horseradish peroxidase-conjugated secondary antibody (Akoya Biosciences, Marlborough, MA, USA). Antigen-expressing cells were detected using Opal fluorophores according to the vendor’s instructions. IHC staining for 8-OHdG was carried out using a primary antibody against 8-OHdG (1:500 dilution; Abcam) and a horseradish peroxidase-conjugated secondary antibody. The slides were then incubated in 3,3′-diaminobenzidine, counterstained with Mayer’s hematoxylin (Novolink; RE7280-K), and mounted with malinol. Standard Masson’s trichrome staining was performed to evaluate the severity of bladder fibrosis. Each whole section was recorded using a digital camera, and color settings and image quantification were determined using image analysis software (ImageJ version 1.51; National Institutes of Health).

### 4.7. mtDNA Copy Number Detection of the Bladder

To measure the mtDNA copy number, the ratio of cDNA amplified from mtDNA-encoded NADH dehydrogenase subunit 1 (ND1) to nucleus-encoded 18S RNA genes was evaluated [49]. The primer sequences used for mtDNA copy number detection were as follows: ND1 Forward (5′-3′) TCGGAGCCC TACGAGCCGTT/Reverse (5′-3′) AGGGAGCTCGATTTGTTTCTG; 18S rRNA, Forward (5′-3′) TAGTTGGATCTTGGGAGCGGG/Reverse (5′-3′) CCGCGGTCCTATTCCATTATT. 18S rRNA served as the control for mtDNA for reaction efficiency. The quantitative real-time polymerase chain reaction (qPCR) was performed in a Roche LightCycler 480 (Roche Applied Science, Mannheim, Germany) apparatus with the Light Cycler 480 SYBR Green I Master kit (Roche Applied Science). The value was determined for each individual qPCR run. ΔCt¼[Ct (ND1) Ct (18S)] represents the relative abundance. The quantitative results were expressed as the copy number of the mtDNA/sample by 2 ΔCt. Each measurement was at least triplicate and normalized in each experiment against serial dilutions of a control DNA sample.

### 4.8. Western Blots for Bladder Proteins

Western immunoblotting was performed using the bladder tissues for ischemia markers, proinflammatory molecules, and fibrosis markers. The procedures were reported previously [23,24]. In brief, alternative samples from each group were homogenized on ice in the CelLytic^TM^MT cell lysis buffer (Sigma-Aldrich, St. Louis, MO, USA) containing a protease inhibitor. The total protein was measured using the Pierce 660 nm protein assay (Thermo, Waltham, MA, USA). Sodium dodecyl sulfate–polyacrylamide gel electrophoresis was performed using the Laemmli buffer system.

Antibodies raised against p-eNOS (ser1175) (1:2000 dilution; Santa cruz, Santa Cruz, CA, USA), VEGF (1:2000 dilution; Abclonal, Woburn, MA, USA), NGF (1: 5000 dilution; cell signal, Danvers, MA, USA), HIF-1α (1:2000 dilution; Abclonal), HIF-2α (1:2000 dilution; Abclonal), AMPKα2 (1:2000 dilution; Abclonal), p-AMPKα2 (1:2000 dilution; Abclonal), ICAM-1 (1: 2000 dilution; Abclonal), NF-κB (1:2000 dilution; Cell Signal), COX-2 (1:2000 dilution; Abcam, Cambridge, UK), IL-1β (1:1000 dilution; Abcam), IL-6 (1:1000 dilution; Abcam), TNF-α (1:2000 dilution, cell signal), PGC-1α (1:2000 dilution; cell signal), TFAM (1:5000 dilution; LSBio, Lynnwood, WA, USA), NRF2 (1:2000 dilution; Abclonal), MnSOD (1:5000 dilution; Cayman, Ann Arbor, MI, USA), CuSOD (1:5000 dilution; Cayman), TGF-β1 (1:5000 dilution; Abclonal), Collagen I (1:1000 dilution; arigo, Hsinchu County, Taiwan), Collagen III (1:1000 dilution; arigo), Fibronectin (1:2000 dilution; BD, Franklin Lakes, NJ, USA), and GAPDH (1:10,000 dilution; Millipore, Burlington, MA, USA) were used.

## 5. Statistical Analysis

All data are presented as the mean ± standard error of the mean (SEM). Data were subjected to a one-way analysis of variance and multiple comparisons using Dunnett’s test. Paired *t*-tests were also used. For all statistical tests, *p* < 0.05 was considered statistically significant.

## Figures and Tables

**Table 1:** General characteristics of experimental animals, *n* = 12, in each group.

Mean ± SEM	Control	Fructose	Fructose/ Tadalafil
**General characteristics:**			
Body weight (g)	290.7 ± 3.4	300.3 ± 3.2	291.3 ± 3.2
Bladder weight (mg)	99.5 ± 1.8	109.7 ± 4	105.8 ± 3.7
Mean arterial pressure (mmHg)	120.2 ± 3.3	148.5 ± 3.4 *	144.1 ± 3.2 *
**Fasting biochemistry parameters**			
Triglycerides (mg/dL)	41 ± 2.5	88.7 ± 4.2 *	57.2 ± 2.2 *
Cholesterol (mg/dL)	67.5 ± 3.5	85 ± 1.8 *	79.2 ± 3 *
Creatinine (mg/dL)	0.74 ± 0.06	0.67 ± 0.06	0.78 ± 0.05
Uric acid (mg/dL)	1.5 ± 0.06	1.9 ± 0.07 *	1.7 ± 0.08 *
Glucose (mM)	5.2 ± 0.11	5.5 ± 0.5	5.2 ± 0.12
Insulin (mU/L)	10.5 ± 1.1	27.9 ± 1.6 *	24.9 ± 1.37 *
HOMA-IR	2.5 ± 0.26	6.8 ± 0.45 *	5.7 ± 0.43 *
Nitrite (μM)	16.6 ± 1.76	33.9 ± 3.8 *	22.3 ± 2.9
Nitrate (μM)	27.3 ± 2.8	21.9 ± 3.6	26.4 ± 5
**Metabolic cage study/24 h**			
Water intake (mL)	43.7 ± 1.6	38.3 ± 2.6	41.8 ± 1.2
Urine output (mL)	22.8 ± 1.9	20.9 ± 1.7	19.3 ± 0.8
No. voids	17.4 ± 1.2	21.1 ± 0.79 *	17.1 ± 0.59
**Cystometric parameters**			
Voiding pressure (mmHg)	26.4 ± 0.96	27.2 ± 0.91	25.8 ± 1.1
Intercontractile interval (min)	12.3 ± 0.5	7.9 ± 0.3 *	11.9 ± 0.6

Data are presented as the mean ± SEM. * indicates significance in comparison with the control group.

## Data Availability

The raw data supporting the conclusions of this article will be made available by the authors on request.
